# Feline Foamy Virus is Highly Prevalent in Free-Ranging *Puma concolor* from Colorado, Florida and Southern California

**DOI:** 10.3390/v11040359

**Published:** 2019-04-19

**Authors:** Sarah R. Kechejian, Nick Dannemiller, Simona Kraberger, Carmen Ledesma-Feliciano, Jennifer Malmberg, Melody Roelke Parker, Mark Cunningham, Roy McBride, Seth P. D. Riley, Winston T. Vickers, Ken Logan, Mat Alldredge, Kevin Crooks, Martin Löchelt, Scott Carver, Sue VandeWoude

**Affiliations:** 1 Department of Microbiology, Immunology, and Pathology, College of Veterinary Medicine and Biomedical Sciences, Colorado State University, Fort Collins, CO 80523, USA; skecheji@colostate.edu (S.R.K.); dannemillern@gmail.com (N.D.); 2 Biodesign Institute, Arizona State University, Tempe, AZ 85281, USA; simona.kraberger@gmail.com; 3 Division of Infectious Diseases, University of Colorado Anschutz Medical Campus, 12700 E 19th Ave, Aurora, CO 80045, USA; Carmen.Ledesma_Feliciano@colostate.edu; 4 Wyoming State Vet Lab, University of Wyoming, 1174 Snowy Range Road, Laramie, WY 82072, USA; jennifer.malmberg@uwyo.edu; 5 Frederick National Laboratory of Cancer Research, Leidos Biomedical Research, Inc., Frederick, MD 21701, USA; melody.roelke-parker@nih.gov; 6 Florida Fish and Wildlife Conservation Commission, 1105 SW Williston Road, Gainesville, FL 32601, USA; mark.cunningham@myfwc.com; 7 Rancher’s Supply Inc., Alpine, TX 79830, USA; livestockprotection@gmail.com; 8 National Park Service, Santa Monica Mountains National Recreation Area, Thousand Oaks, CA 90265, USA; seth_riley@nps.gov; 9 Karen C. Drayer Wildlife Health Center, University of California, Davis, CA 95616, USA; twvickers@ucdavis.edu; 10 Wildlife Researcher Colorado Parks and Wildlife, 2300 S. Townsend Avenue, Montrose, CO 80203, USA; ken.logan@state.co.us; 11 Colorado Division of Wildlife Office, Mammals Research, 317 W. Prospect Rd, For Collins, CO 80526, USA; Mat.alldredge@state.co.us; 12 Department of Fish, Wildlife, and Conservation Biology, Colorado State University 115 Wagar, Fort Collins, CO 80523, USA; kevin.crooks@colostate.edu; 13 Department of Molecular Diagnostics of Oncogenic Infections, Research Program Infection, Inflammation and Cancer, German Cancer Research Center, (Deutsches Krebsforschungszentrum Heidelberg, DKFZ), Im Neuenheimer Feld 242, 69120 Heidelberg, Germany; m.loechelt@dkfz-heidelberg.de; 14 School of Biological Sciences, University of Tasmania, Sandy Bay, Tasmania 7005, Australia; Scott.carver@utas.edu.au

**Keywords:** feline foamy virus, epidemiology, retrovirus, *Spumaretrovirus*, mountain lion, *Puma concolor*, ELISA

## Abstract

Feline foamy virus (FFV) is a retrovirus that has been detected in multiple feline species, including domestic cats (*Felis catus*) and pumas (*Puma concolor*). FFV results in persistent infection but is generally thought to be apathogenic. Sero-prevalence in domestic cat populations has been documented in several countries, but the extent of viral infections in nondomestic felids has not been reported. In this study, we screened sera from 348 individual pumas from Colorado, Southern California and Florida for FFV exposure by assessing sero-reactivity using an FFV anti-Gag ELISA. We documented a sero-prevalence of 78.6% across all sampled subpopulations, representing 69.1% in Southern California, 77.3% in Colorado, and 83.5% in Florida. Age was a significant risk factor for FFV infection when analyzing the combined populations. This high prevalence in geographically distinct populations reveals widespread exposure of puma to FFV and suggests efficient shedding and transmission in wild populations.

## 1. Introduction

Feline foamy virus (FFV) is a member of the oldest retrovirus family, *Spumaretrovirinae* [1]. The virus is reportedly contact-dependent and causes life-long infections in felines worldwide [2]. FFV was originally identified as a tissue culture contaminant from primary feline cell cultures [3] and named for its characteristic cytopathic effects. In comparison to other feline retroviruses, the relevance of FFV infection to felid behavior and health is not yet well-understood despite its high prevalence in populations worldwide [4]. Existing epidemiological studies on FFV have almost exclusively evaluated domestic cat (*Felis catus*) populations [5,6], with only a handful evaluating prevalence in wild feline species [7,8,9,10]. Additionally, published literature has found FFV to be putatively apathogenic in domestic cats [11] but no literature has explored the virus’ relationship to pathology in wild felids. Puma (*Puma concolor*) are the largest felid in North America and have frequent contact (e.g. predation) with domestic cats [12], making them a unique subject for this analysis.

In this study we exploited an extensive archive [13] to investigate FFV sero-prevalence and risk factors in pumas in the United States using a serologic assay validated for use in domestic cats [14,15,16]. Relationships between puma demography (e.g. age and sex) and FFV infection was determined using a Bayesian hierarchical modeling approach. We additionally explored the effect of a regional treatment (sport-hunting ban) on FFV sero-positivity in one Colorado subpopulation in order to investigate the effect of management interventions on the spread of the virus. Our results suggested that FFV sero-prevalence in U.S. puma was high (78.6% overall), risk factors varied by sampling location, and that a ban on hunting did not affect FFV sero-prevalence in Colorado.

## 2. Materials and Methods

We evaluated FFV sero-prevalence in three states: Colorado (*n* = 130, collected 2005–2011), Florida (*n* = 150, collected 1983–2010), and Southern California (*n* = 68, collected 2001–2011). Puma samples were opportunistically collected by government and local authorities engaged in independent management studies as previously described [13,17]. Sex and age of sampled pumas, if recorded, were determined via manager expertise, categorized as male or female and adult or young. Due to inconsistent location information, each state’s sample population was considered a uniform population.

Sera were tested in duplicate on separate 96-well plates at 1:50 dilution using a non-quantitative GST-capture ELISA targeting the FFV Gag antigen, as previously described [14,15,16]. The ELISA has high sensitivity and specificity for the detection of FFV antibodies in naturally and experimentally infected domestic cats and has been validated against western blot [14,15,16]. This ELISA utilizes recombinant FFV Gag antigen generated from domestic cat FV sequences, which are 98% similar to the published puma FFV Gag [18,19]. Additional data evaluating more than 50 *gag* sequences from Colorado pumas has determined 95–100% similarity between puma and domestic cat FFVs [20]. A positive ELISA result was defined as having an OD absorbance over [2 × (meanGag + 3 SD)], with “meanGag” being the average negative control absorbance, and “SD” being the standard deviation of the negative control absorbance. This calculation employs very stringent cut off criteria [14,15,16], and is similar to methods of analytical detection reported by Lardeux et al. (2016) [21]. OD calculation was revised for each run, assuring each analysis was compared to its own negative control. Serum from experimentally infected domestic cats (positive) or specific pathogen free domestic cats (negative) were used as controls [15]. All plates were run in the same laboratory by the same individual over the course of one month. Reagents were reconstituted on an as-needed basis. ELISA plates were prepared with fresh coating buffer the night prior to use.

Normalization of results was conducted by calculating each sample’s absorbance as a percent of average positive control absorbance on the same plate. Each sample was additionally recorded as positive or negative using OD cut off values calculated as described above.

FFV sero-prevalence across the sampling period, stratified by sex (male and female) and age (young and adult) across all locations was compared using a chi-square test. Sex, age, and the interaction between sex and age were evaluated as possible risk factors for FFV infection for each state and across all samples using Bayesian generalized linear models (GLMs, a style of linear regression accounting for response variables with non-normal error distributions). For each coefficient (i.e., variable), we used weakly informative priors and extracted a 95% credible interval from the posterior distribution. Any coefficient whose 95% credible interval did not contain 0 was considered important. GLMs were ranked and compared using Akaike information criterion (AIC, an estimator of the relative quality of a statistical model when compared to other models for a given set of data). The model with the lowest AIC value was considered to better fit the data and subsequently the important variable(s) within that model were considered potential risk factors for puma FFV infection. If a model had an important predictor and was within 2 AIC of the best fit model, it was considered to reveal the most credible risk factor for FFV infection in pumas. Puma with unrecorded sex (*n* = 10) or age (*n* = 74) were excluded from risk factor analyses. There were 173 female, 167 male, 190 adult and 86 young pumas used in the analysis.

We additionally evaluated FFV sero-prevalence during and after a management intervention in Colorado. Between 2004 and 2009, Colorado Parks and Wildlife implemented a sport hunting ban in the Western Slope to evaluate management programs [22]. We compared FFV sero-prevalence during (2004–2009) and after (2010–2014) the sport hunting ban using a chi square statistic.

## 3. Results

### 3.1. Sero-Prevalence

**Figure 1:**
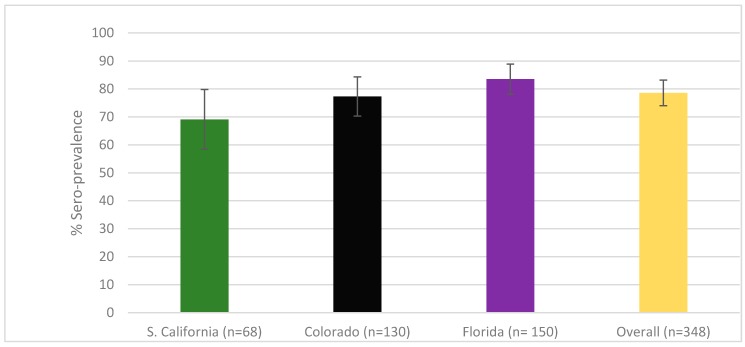
Puma feline foamy virus was found in high sero-prevalence in the U.S. Sero-prevalence by state: Southern California 69.1% (95% CI: 56.7, 79.8%); Colorado 77.3% (95% CI: 69.1, 84.3%); Florida 83.5% (95% CI: 77.0, 88.9%). Sero-prevalence across all sampling locations (overall) was 78.6% (95% CI: 74.0, 82.7%). There was no significant difference between states (*p* = 0.14).

FFV sero-prevalence is reported in Figure 1 and was high in all three states with an overall sero-prevalence of 78.6% (95% CI: 74, 82.7). There was no significant association of FFV sero-prevalence with location at the state level.

**Figure 2:**
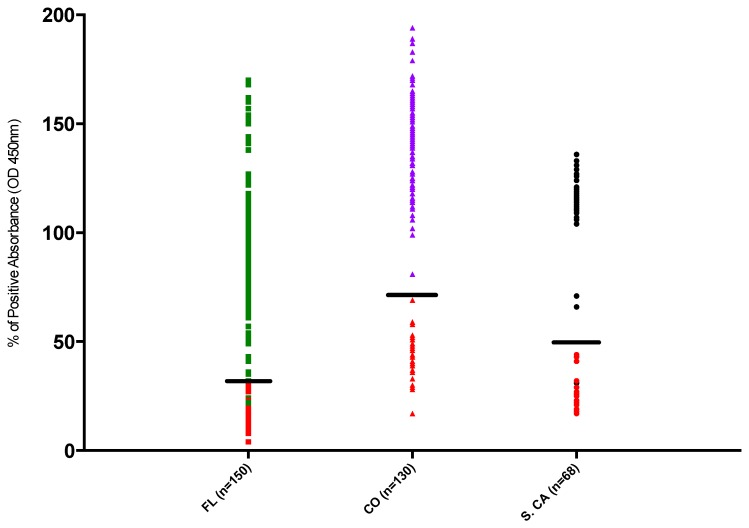
Seropositive OD values were well distinguished from samples classified as seronegative. The sample points in red were negative; the sample points in green, purple or black were classified as positive for their respective locations. All Colorado and all but one Southern California sample fell into positive absorbance ranges that were clearly segregated (noted with black lines) from samples classified as negative (<70% for CO, <50% for S.CA). 112 of 119 Florida samples classified as seropositive were at least 49% of positive control OD values, with only two positive samples <30% (noted with black line).

Figure 2 displays the normalized distribution of all samples used in this analysis, grouped by location, and colored by negative and positive assignment. Colorado samples with normalized absorbance >70% of the positive control values were consistently classified as positive, while samples <70% of the positive control’s absorbance were negative. Southern California samples classified as positive had absorbance values >50% of the positive control absorbance except for one sample that was classified 31% of positive control value but still above negative cut off for the plate. All other Southern California samples classified as negative had absorbance <50%. Colorado and Southern California samples classified as positive were clearly distinguished by negative cut off values (Figure 2). While there was less demarcation of clearly negative and positive samples in the Florida population, only two positive samples were <30% absorbance and all negative samples were <30%. Reclassification of all Florida panthers with an absorbance less than 49% (*n* = 7) as negative would have resulted in a sero-positivity of 74% which fell outside of the calculated 95% confidence interval (estimated as 77.0–88.9%); however, exclusion of these samples would have resulted in a sero-positivity of 78%, which remained within the calculated 95% confidence interval (estimated as 77.0–88.9%).

### 3.2. Demographic Associations

**Figure 3:**
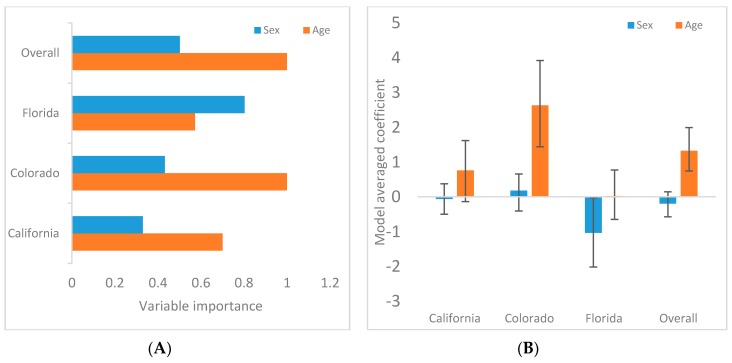
Older pumas and female Florida panthers at higher risk of feline foamy virus (FFV) infection. (**A**) The plot displays variable importance weights for puma sex or age as risk factors for FFV, showing age to be more important in Colorado, Southern California and across the sampling locations (overall). (**B**) The plot displays model averaged coefficients with 95% confidence intervals, with adult and male being >0, and young and female being <0. If the 95% confidence interval did not cross 0, the variable was considered important. Females were weakly associated with infection risk in Florida only.

Results of Bayesian GLMs are reported and graphically portrayed in Table 1 and Figure 3, respectively. Neither sex nor age were predictors of FFV infection in Southern California, but there was a trend for higher FFV exposure in adults relative to younger pumas. Age was a predictor for FFV infection over all sites and also in Colorado and Southern California as individual sites, with adult pumas being at greater risk. In Florida as an individual site, sex, not age, was a predictor of FFV infection, with females being at greater risk.

There was no significant difference in FFV sero-prevalence during (*n* = 40) and after (*n* = 85) the sport hunting ban implemented on the Western Slope of Colorado (X^2: 2.5, *p* > 0.11).

## 4. Discussion

This study provides the first broad scale investigation of FFV sero-prevalence in a large, geographically-dispersed sampling of free-ranging pumas. The high overall puma FFV sero-prevalence of 78.6% was higher than what had been recorded in domestic cat populations using similar ELISA assays [5,6]. Reported FFV infection rates in domestic cats have ranged from 30 to 70%, and have varied across locations and analytical methodology [2,5,6,7,15]. Although the puma sampling locations have marked differences in their landscapes and ecology [13] similarly high infection rates were detected in all populations. This suggests that FFV is readily transmitted across varied landscapes.

While it was not possible to validate this study using sera from known positive and negative individuals, our stringency for negative and positive values was set using strict criteria [21]. Analysis of OD values illustrated distinct clustering of positive and negative sample absorbance (Figure 2), providing confidence that positive values truly reflected actual sero-prevalence. Florida samples were not as clearly distinguished as Colorado and Southern California samples. Samples from Florida were typically older and more likely to have been collected under less-than-ideal conditions from autolyzed animals (i.e., from road kill specimens). While this would typically result in the detection of more false negatives than false positives (due to degradation of circulating antibodies) it was also possible that there was higher nonspecific binding in this cohort. While exclusion of positive samples falling below 50% OD values of positive controls would still result in sero-prevalence estimates within the estimated 95% confidence interval in this population, further confirmatory analysis by PCR or other serologic assay would be warranted.

The significant difference in sero-prevalence between age groups in Colorado and across the sampled locations suggests that adults have higher exposure to FFV. If adult pumas are at increased risk of FFV infection, it is necessary to further investigate FFV transmission and the potential for horizontal transmission between individuals being the dominant mode. The significantly lower sero-prevalence of FFV in kittens suggests that this age group does not have the same exposure to the virus as adults in the same populations, supporting horizontal transmission as the primary mode of transmission in these populations. We recognize the difficult task of sampling family units is needed to fully explore the possibility of vertical transmission of the virus; however, a future study with this goal could help to more clearly determine mother to cub transmission events.

This reported high FFV sero-prevalence in adults exceeded the prevalence of most other infectious agents reported in these populations [13]. Adult pumas are traditionally considered to be solitary animals with rare documented instances of geographic and temporal overlap [23,24,25] though recent behavioral studies have exposed more social interactions between pumas [26]. In either case, inferred horizontal transmission of FFV resulting in four of five adults being exposed suggests facile transmission among adults. High FFV sero-prevalence could be explained by the virus maybe being able to survive on fomites (e.g., carcasses) long enough to infect other pumas without the need for physical contact between pumas. Further investigation of FFV degradation outside the host is needed to explore this alternative hypothesis. Further investigation of the virus’ genetic and biological attributes may provide unique insights into puma behavior and capacity for transmission of other pathogens similar to Fountain-Jones et al. (2018) [27].

The lack of significant difference in sero-prevalence between sex groups in Southern California, Colorado and overall is consistent with what we found in domestic cats [28]. This suggests transmission may be via non-antagonistic intraspecific interactions. Physical contacts during mating or saliva swapping during kill sharing may be the most likely types of primary transmission events, as there is no substantial evidence that prolonged amicable physical contact (e.g., grooming) is common between adults [23,24,26]. A recent report has documented higher FFV loads in saliva versus blood in naturally infected domestic cats [29], which could suggest a mechanism for puma to puma transmission. Further studies of FFV ecology in the endangered and federally protected Florida panther (Puma concolor coryi) are needed to further our understanding of female sex as a risk factor for FFV infection.

FFV has not been documented to cause acute disease or easily recognized clinical signs of disease in domestic cats [2,14,30]. However, simian Foamy Virus (SFV) has been associated with accelerated SIV disease [31] and an association between FFV viral load and exogenous Feline leukemia virus (FeLV) viremia has been noted [31,32]. Therefore, results from this study can be used to compare the prevalence of FFV to other retroviruses of pumas such as feline immunodeficiency virus (FIV) and evaluate possible potentiation of disease. Furthermore, since FFV is a lifelong infection and viral sequences can be isolated from circulating blood cells, genotypic analysis of puma FFV may be useful as a marker of animal movement and pathogen transmission within or between populations [33].

## Figures and Tables

**Table 1:** Age alone as a risk factor for feline foamy virus (FFV) infection produced the best fit model in Southern California, Colorado and across all sampling locations (overall), while in Florida sex was the risk factor that produced the best fit model. Models were ranked from most to least supported by the data.

Model	AIC	ΔAIC	Model Weight
**SOUTHERN** **CALIFORNIA**			
Age	63.39	0.00	0.46
Null	64.98	1.59	0.21
Sex + Age	65.34	1.94	0.18
Sex	66.7	3.38	0.09
Sex + Age + Sex*Age	67.34	3.94	0.06
**COLORADO**			
Age	62.09	0.00	0.57
Sex + Age	63.87	1.78	0.23
Sex + Age + Sex*Age	64.20	2.11	0.20
Null	77.69	15.60	0.00
Sex	79.23	17.14	0.00
**FLORIDA**			
Sex	111.53	0.00	0.32
Sex + Age + Sex*Age	111.77	0.24	0.29
Sex + Age	112.55	1.02	0.19
Null	113.77	2.24	0.11
Age	114.02	2.49	0.09
**OVERALL**			
Age	240.22	0.00	0.50
Sex + Age	240.84	0.62	0.37
Sex + Age + Sex*Age	242.83	2.61	0.14
Sex	254.40	14.18	0.00
Null	256.12	15.90	0.00
